# Diffuse Pulmonary Meningotheliomatosis: An Exceedingly Rare Disease for a Not-So-Rare Pattern

**DOI:** 10.7759/cureus.87023

**Published:** 2025-06-30

**Authors:** Marta Monteiro de Castro, Luís Lázaro Ferreira, Agostinho Sanches, Sérgio Campainha

**Affiliations:** 1 Pulmonology Department, Unidade Local de Saúde Gaia/Espinho, Vila Nova de Gaia, PRT; 2 Pathology Department, Unidade Local de Saúde Gaia/Espinho, Vila Nova de Gaia, PRT

**Keywords:** diffuse pulmonary meningotheliomatosis, interstitium, meningothelial-like cells, pulmonary biopsy, pulmonary micronodules

## Abstract

Diffuse pulmonary meningotheliomatosis (DPM) is a rare lung disease characterized by the proliferation of meningothelial-like cells within the pulmonary interstitium. It predominantly affects middle-aged women and is often asymptomatic, although mild respiratory symptoms may occur. Chest CT typically shows diffuse and bilateral micronodules, and histology confirms meningothelial-like nodules expressing epithelial membrane antigen (EMA), CD56, and progesterone receptor. We report the case of a 72-year-old woman undergoing breast cancer staging, who was incidentally found to have bilateral pulmonary micronodules. A conventional transbronchial biopsy confirmed DPM, and a brain MRI ruled out meningioma. The patient remains stable without treatment. DPM is often misdiagnosed, with metastatic disease and granulomatous conditions as key differentials. Although biopsy aids diagnosis, correlation with imaging findings is essential. No specific treatment is required, and the prognosis is generally favorable. Further research is needed to refine diagnoses and management strategies.

## Introduction

Diffuse pulmonary meningotheliomatosis (DPM) is a rare pulmonary disease characterized by the proliferation of meningothelial-like cells within the pulmonary interstitium. These cells share the morphological and immunohistochemical profiles with those of meningiomas. DPM is most frequent in females and is more commonly detected between the fifth and sixth decades of life. Most patients are asymptomatic, but some can present some mild, nonspecific symptoms, such as dyspnea and dry cough. On chest CT, DPM is characterized by diffuse and bilateral ground-glass or solid micronodules (<6 mm in diameter) randomly distributed throughout the lungs. Some nodules may feature a central lucency (“cheerio” sign). This micronodular pattern appears in other diseases such as granulomatous processes or pulmonary metastases, which is why a lung biopsy is essential for accurate diagnosis. DPM is characterized by minute meningothelial-like nodules (MMNs), which are benign lesions composed of small nests of epithelioid cells expressing meningothelial cell markers (epithelial membrane antigen (EMA), CD56, and progesterone receptor (PR)). These types of nodules can be incidental findings in lung biopsies performed for various reasons, including diagnoses of neoplastic or interstitial diseases, and their significance is uncertain. The diagnosis of DPM implies a correlation between the chest CT image and the absence of identification of other lesions as the cause of the micronodules [1,2].

## Case presentation

The authors present a case of a 72-year-old nonsmoker, a Caucasian woman without relevant occupational or environmental exposures. Her past medical history included a recent diagnosis of carcinoma of the left breast under neoadjuvant therapy with letrozole and awaiting surgery; arterial hypertension under an angiotensin receptor antagonist; dyslipidemia under statins; and supplemented hypothyroidism. Regarding surgical history, she underwent appendectomy, tonsillectomy, hysterectomy, and bilateral adnexectomy (for benign disease), and orthopedic surgery on the right shoulder. She had no family or personal history of pulmonary or autoimmune disease.

**Figure 1:**
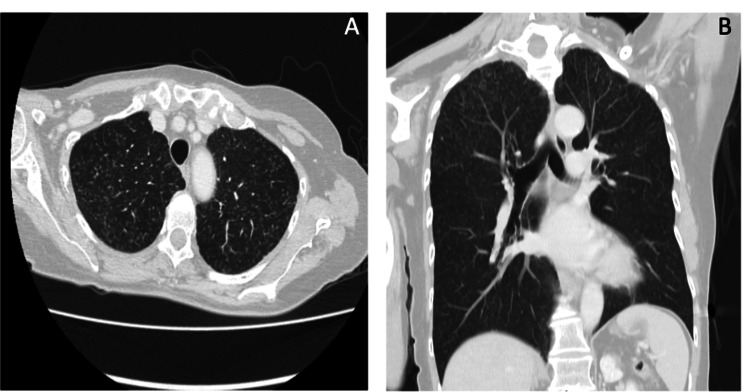
Chest CT images A and B: Chest CT images (axial and sagittal lung window). Multiple and bilateral ground-glass micronodules in a random distribution.

In a chest CT for breast cancer staging, bilateral micronodules were detected, with a random distribution and predominance in the upper lobes (Figure 1).

**Figure 2:**
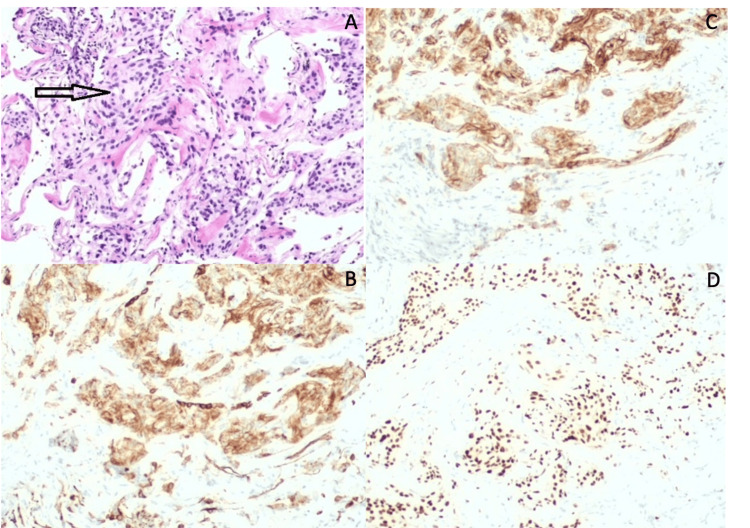
Histopathological examination Histopathological examination revealed patches of lung tissue with focal proliferation of oval cells, in aggregates, without atypia, mitotic or necrotic figures, with septal thickening. Lung biopsy with discrete proliferation of epithelioid and oval cells, without atypia or mitotic figures (A). In the immunocytochemical study, positivity was observed for CD56 (B), epithelial membrane antigen (C), and progesterone receptors (D).

Clinically, the patient had only dyspnea, modified Medical Research Council grade 1 (mMRC 1). She had no symptoms suggestive of autoimmune disease. Physical examination was unremarkable. Given the radiological changes, it was decided to continue the investigation with lung function tests and bronchoscopy for microbiological samples and conventional transbronchial lung biopsy. Lung function tests only revealed a mild diffusion defect (transfer factor of the lung for carbon monoxide (TLCO) 62% of predicted). Microbiological and mycobacterial tests from bronchial lavage were negative. Histopathological examination revealed morphological and immunohistochemical aspects compatible with MMNs (EMA, CD56, and PR positive) (Figure 2).

Given the findings, a brain and neuro-axis MRI was performed, which excluded meningioma. The clinical, radiological, and pathological findings supported the diagnosis of pulmonary meningotheliomatosis in a multidisciplinary discussion. The patient remains stable at one year without any specific therapy.

## Discussion

In this clinical case, considering the clinical context and the micronodular pattern detected on chest CT, the main differential diagnoses raised were a granulomatous process (sarcoidosis or infectious) or metastatic disease. Sarcoidosis was a diagnostic hypothesis, as the micronodular pattern was compatible with the diagnosis. However, the patient did not present any other suggestive symptoms, and the biopsy did not support this diagnosis. The infectious process was also ruled out after obtaining negative microbiological results and the biopsy result. Given the personal history of breast cancer, pulmonary metastasis was also a differential diagnosis, which was excluded through transbronchial biopsy. The diagnosis of DPM, due to its rarity, was not considered; however, after clinical, radiological, and histological integration, it was the final diagnosis. The “cheerio sign” on chest CT, although not pathognomonic, may constitute a helpful diagnostic feature, even though it was not present in our case. Biopsy plays an essential role in the differential diagnosis of this entity, yet surgical lung biopsy is not mandatory, as demonstrated in our case. To confidently diagnose DPM, the isolated findings of MMNs in lung biopsy or a micronodular pattern on chest CT are not enough, and it is necessary to combine the two factors. Considering that the lung is the most common site for metastasis of meningiomas, it is also necessary to exclude the possibility of metastatic deposits of meningioma. In terms of treatment, no specific treatment is indicated for this disease, and the prognosis is usually good, with prolonged stable disease without any therapy [1]. There are few cases reported in the literature, with variations, for example, in the type of biopsy used, including surgical biopsy [3-5], transbronchial lung cryobiopsy (TBC) [6], and transbronchial biopsy [7]. In the clinical case presented, a conventional transbronchial biopsy was sufficient for diagnosis; however, if it had not been, it might have been necessary to proceed with techniques that allow obtaining larger fragments, namely, TBC or surgical biopsy, as in other cases described in the literature.

Information about performing a brain and neuro-axis MRI to exclude meningioma, which the authors consider of paramount importance, is lacking in most cases [3-7]. There is no mention of any specific treatment for this disease, but one case report refers to the suspension of hormone replacement therapy due to potential hormonal influence [4]. Therefore, the approach is conservative, and case reports refer to stability over the years, with some cases demonstrating stability for at least two years [4-7]. The patient described in our clinical case remains under surveillance, with clinical, imaging, and functional stability.

## Conclusions

DPM should be considered a differential diagnosis in patients presenting with a nonspecific, diffuse micronodular pattern. The correlation between histological and imaging findings is essential for accurate diagnosis. Exclusion of meningioma is also crucial, as the lungs represent the most common site of meningioma metastasis. Current knowledge about this condition remains limited, and further research is needed to understand why some patients exhibit only sparse MMNs on histology, while others develop a sufficient burden to be detectable on imaging.
